# Optimizing Achilles Tendon Rupture Care: The Efficacy of Physiotherapy-Led Conservative Management in a District General Hospital

**DOI:** 10.7759/cureus.75657

**Published:** 2024-12-13

**Authors:** Abdelwakeel Bakhiet, Annika Lakhani, Abdullah Bin Sahl, Khadija Elamin, Yousof Marjan, Anand Pillai

**Affiliations:** 1 Trauma and Orthopaedics, Wythenshawe Hospital, Manchester, GBR; 2 Trauma and Orthopaedics, Leeds Teaching Hospital, Leeds, GBR; 3 Trauma and Orthopaedics, Royal College of Surgeons in Ireland, Dublin, IRL; 4 Trauma and Orthopaedics, Manchester University NHS Foundation Trust, Manchester, GBR; 5 Trauma and Orthopaedics, Stepping Hill Hospital, Manchester, GBR; 6 Trauma and Orthopaedics, Wythenshawe Hospital, Manchester University NHS Foundation Trust, Manchester, GBR

**Keywords:** achilles rupture, achilles tendon injury, achilles tendon rupture, functional rehab, physiotherapy

## Abstract

Introduction

Achilles tendon rupture (ATR) represents a significant musculoskeletal injury that can affect many patients' mobility and quality of life. Treatment of ATR consists of both conservative and surgical options, with the traditional belief being that surgical intervention reduces the risk of re-rupture. However, with the introduction of physiotherapy-led functional rehabilitation strategies with early mobilization, it has been shown that re-rupture rates are equal among surgical and non-surgical patients. This study focuses on evaluating the effectiveness of non-surgical, physiotherapy-led management, specifically within the context of patients at a district general hospital. The rationale for investigating this approach lies in the growing interest and adoption of conservative management strategies for ATR across orthopaedic practices within the National Health Service (NHS).

Methods

This is a retrospective cross-sectional study assessing the efficacy of the physiotherapy-led treatment pathway in our district general hospital. Data were retrospectively collected from Hive, which is the trust’s electronic patient record (EPR) and has comprehensive access to all patients' history, notes, images, investigations, and previous letters. We looked at all patients treated by physiotherapists for ATR from September 2022 to December 2023.

Results

In total, 76 patients were treated along the physiotherapy-led pathway for ATR between September 2022 and December 2023. The ages ranged from 22 to 82 years, with a mean age of 47 years. Of the patients, 48.6% (n = 37) were in the 40-60 years age range. The gender distribution was 69.7% (n = 53) males and 30.3% (n = 23) females (total = 76). The injury characteristics revealed a predominance of complete ATR, with 88.1% (n = 67) of cases being complete ATR, and 11.9% (n = 9) being partial ATR. Patients experiencing re-ruptures ranged from 34 to 64 years old, with three males and one female. Re-escalation to a consultant in only 14.5% of cases (n = 11) reflects successful initial management, minimizing the need for further interventions. Based on the available data, on average, younger patients (≤49 years) returned to work approximately 5.7 weeks post injury (n = 32). Conversely, older patients (above the average age) took 13.5 weeks on average to return to work. The dataset revealed that, on average, patients under the physiotherapy-led pathway returned to sports activities in about 29.9 weeks. Thromboprophylaxis was prescribed for 93.5% (n = 71) of patients with ATR. The data revealed a low incidence rate of deep vein thrombosis with only 4% (n = 3) of patients experiencing this complication post rupture.

Conclusion

This audit offers insight into physiotherapy-led ATR management at Wythenshawe Hospital, evaluating treatment results and challenges. The findings suggest that a conservative approach is effective in promoting patient recovery, with low re-rupture rates and successful return-to-function outcomes. While limitations such as sample size and retrospective design exist, the findings underscore the need for tailored rehabilitation protocols and continued research to optimize patient care in ATR management.

## Introduction

Achilles tendon rupture (ATR) represents a significant musculoskeletal injury that can affect patients' mobility and quality of life. The Achilles tendon forms the tendinous part of the gastrocnemius and soleus muscles where they insert into the calcaneal tuberosity [1]. Over time, the incidence of ATR has reportedly risen [2]. This is often thought to be due to the ageing population and an increase in obese patients, as well as an increase in engagement with sporting activities by middle-aged people [3].

Most injuries occur during athletic activity and the ATR tends to occur within 3-6 cm proximal to the insertion of the Achilles tendon on the calcaneum [4]. Partial tears are very rare, and the ruptures are usually complete and occur without any preceding symptoms but with distinct ankle trauma [5]. This has been described by three different mechanisms of injury. The first is due to pushing off the floor with the forefoot and the knee extended; the second is a sudden force of dorsiflexion on the ankle; and the third is a forceful dorsiflexion of a plantarflexed foot [2].

ATR can be missed on initial presentation in up to 25% of patients [6]. Patients often present with a short acute history of sudden pain in the posterior aspect of their affected leg, and they often describe feeling as if they had been kicked or even shot in the back of the leg or will feel a sudden giving way feeling when pushing off with their heel [7]. Pain resolves quickly; therefore, the patient may not report pain during the examination of the Achilles tendon [8].

The clinical examination consists of an examination of the patient's gait, local swelling, tenderness, and weakness of plantarflexion in comparison to the unaffected side [9]. The most common diagnostic clinical test is the calf-squeeze test, described by Simmonds and Thompson [10]. This is performed by positioning the patient prone on an examination table with their ankle hanging off the end of the table and feet freely hanging. If there is no or minimal plantarflexion in comparison to the unaffected side, then the test is deemed as positive, whereas if plantarflexion is present, then it is negative [11].

ATR is often a clinical diagnosis; however, when clinical findings are equivocal, then ultrasound and MRI can be used to investigate. However, as per the American Academy of Orthopaedic Surgeons (AAOS), they have been described as inconclusive due to a lack of significant evidence [12]. The literature shows that clinical examination with Simmonds' calf squeeze test has a higher sensitivity in detecting acute ATR than radiological investigation.

Treatment of ATR consists of both conservative and surgical options, with the traditional belief being that surgical intervention reduces the risk of re-rupture. However, with the introduction of physiotherapy-led functional rehabilitation strategies, a meta-analysis has shown that re-rupture rates are equal amongst surgical and non-surgical patients, with the Achilles Tendon Total Rupture Score (ATRS) being similar for both patient groups [13]. Functional rehabilitation has been proven as a way to reduce re-rupture rates and avoid the complications associated with surgical treatment such as wound healing complications and infection [14].

Since 2020, the National Health Service (NHS) has faced significant challenges in managing patient load post pandemic, including those with musculoskeletal injuries such as ATR. This may be attributed to various factors such as altered working environments and delayed access to healthcare services due to pandemic-related disruptions [15]. This increased demand for healthcare services, coupled with the backlog of musculoskeletal cases, highlights the urgent need for innovative solutions to alleviate the burden on NHS hospitals.

In our hospital, patients with a suspected ATR are seen in the accident & emergency department or are referred in by general practitioners in the surrounding area. They are assessed by the Simmonds' calf squeeze test and if positive, they are referred directly to the virtual fracture clinic (VFC) and are reviewed within three days by a VFC orthopaedic consultant. If there is any clinical dubiety, then an ultrasound scan is used to confirm the diagnosis. Patients are then referred for a physiotherapy appointment within four weeks where they are guided through the weight-bearing exercises and followed up with the physiotherapy team.

This study focuses on evaluating the effectiveness of non-surgical, physiotherapy-led management, specifically within the context of patients at a district general hospital. The rationale for investigating this approach lies in the growing interest and adoption of conservative management strategies for ATR across orthopaedic practices within the NHS.

## Materials and methods

This is a retrospective cross-sectional study assessing the efficacy of the physiotherapy-led treatment pathway in our district general hospital (DGH). Data were retrospectively collected from Hive, which is the trust’s electronic patient record (EPR) and has comprehensive access to all patients' history, notes, images, investigations, and previous letters [16]. The data were extracted and cleaned, ensuring de-identification and addressing missing values.

When a patient is seen with a suspected or confirmed ATR, then they are referred to the VFC where their case is assessed by a consultant within 48 hours. Fit and active patients below the age of 40 are offered surgery and if they are keen, then they are seen in a consultant-led clinic for further discussion and review. If they are not suitable or decline surgery, then they are treated along the physiotherapy-led pathway. Patients who are above the age of 40 or who are not active are referred to physiotherapy immediately. We included all patients treated by physiotherapists for ATR from September 2022 to December 2023. Patients with confirmed ATR but who were not treated by physiotherapists were not included in the study.

The collected data encompassed a variety of parameters, including the type of rupture (partial or total), the time from injury to the first physiotherapy session, the number of physiotherapy sessions within the initial 12 weeks post injury, and the time taken for patients to return to driving, work, and sports. The occurrence of any re-ruptures, cases where patients were referred to orthopaedic consultants for further management, and the prophylaxis and incidence of any deep vein thrombosis (DVT) were also recorded.

Data were collated within Microsoft Excel version 16.0 for Windows (Microsoft Corporation, Redmond, WA). We generated master tables, including the data points of interest, which were age, gender, date of injury, severity of injury, time from referral to review, time to return to work and driving, and the need for consultant review. The data were analysed using Microsoft Excel and were used to assess the effectiveness of the physiotherapy-led treatment regime for ATR patients.

## Results

In total, 76 patients were treated along the physiotherapy-led pathway for ATR between September 2022 and December 2023. The ages ranged from 22 to 82 years, with a mean age of 47 years. Of the patients, 48.6% (n = 37) were in the 40-60 years age range. The gender distribution was 69.7% (n = 53) males and 30.3% (n = 23) females (total = 76). The average age of male patients was 48.77 years, slightly lower than females whose average age was 50.64 years (Table 1).

**Table 1:** Demographics of patients presenting with Achilles tendon rupture.

Age (years)	N
20-39	21
40-59	37
60-80	15
>80	3
Mean age	49.2
Gender	N
Male	53
Female	23

**Figure 1:**
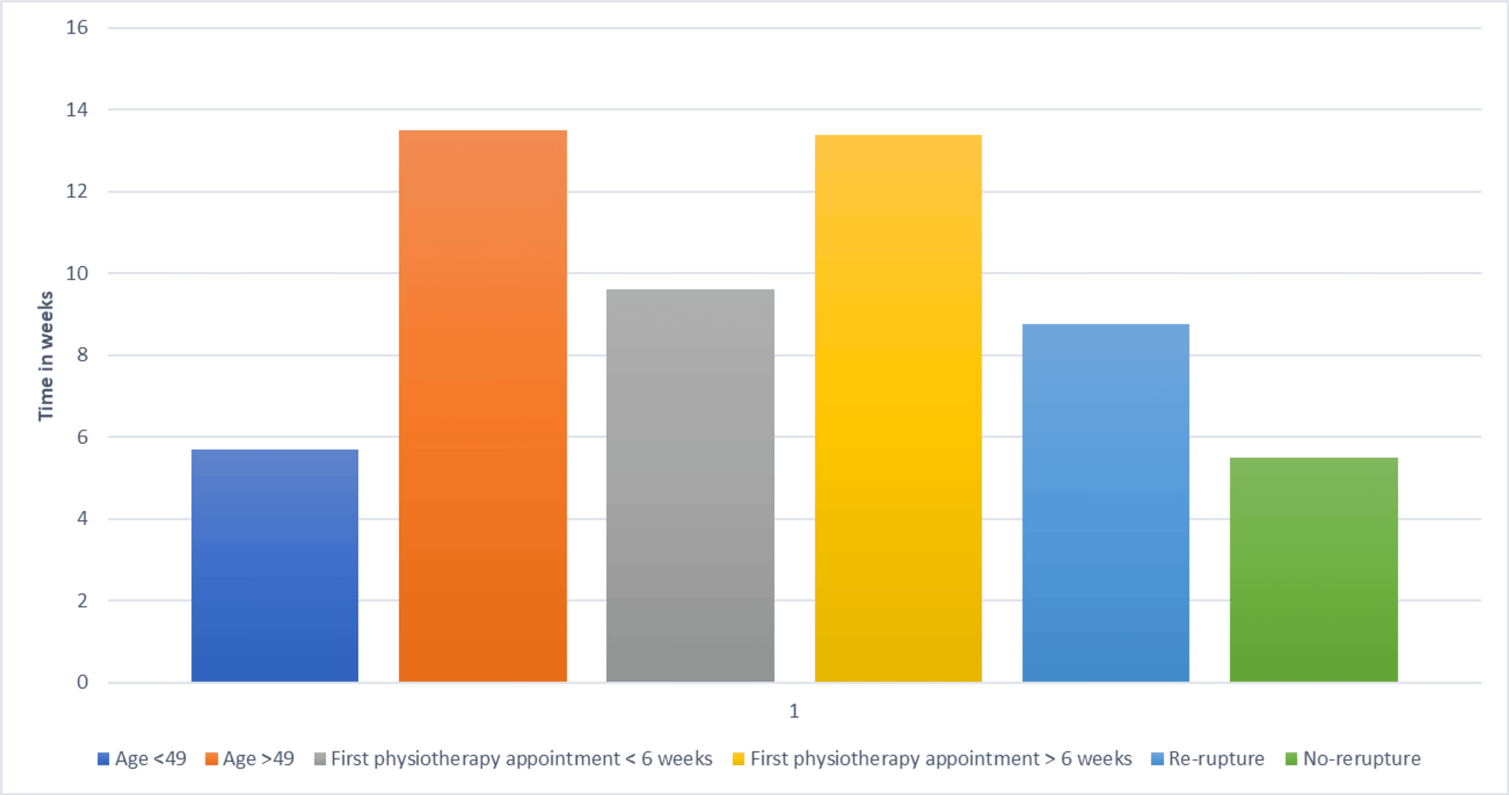
Bar chart showing the effect of age, earlier physiotherapy review, and re-rupture on patients returning to work.

The injury characteristics revealed a predominance of complete ATR, with 88.1% (n = 67) of cases being complete ATR, and 11.9% (n = 9) being partial ATR. The data showed a higher incidence of ruptures on the left side (n = 49) compared to the right (n = 27), suggesting a need for further investigation into possible underlying biomechanical or lifestyle factors contributing to this distribution (Figure 1).

Among the patients analysed, 95% (n = 72) experienced no further ruptures, indicating a 5% (n = 4) re-rupture rate. This underscores the effectiveness of treatment and rehabilitation in minimizing the risk of injury recurrence. Patients experiencing re-ruptures ranged from 34 to 64 years old, with three males and one female. Re-escalation to a consultant in only 14.5% (n = 11) of cases reflects successful initial management, minimizing the need for further interventions.

The data analysis of patients who underwent physiotherapy following ATR highlighted several factors influencing the return to driving. It was observed that 61.8% (n = 47) of patients were able to resume driving after an average of 10 weeks post injury. The severity of rupture influenced return-to-driving time, with complete tendon ruptures necessitating longer recovery periods than partial tears. Specifically, individuals with complete ruptures required a mean of 11.3 weeks to return to driving.

Based on the available data, on average, younger patients (≤49 years) returned to work approximately 5.7 weeks post injury (n = 32). Conversely, older patients (above the average age) took 13.5 weeks on average to return to work. The mean waiting time for the initial physiotherapy session was seven weeks. Patients who were seen within six weeks returned to work in a mean of 9.6 weeks (n = 28), whereas those seen more than six weeks post injury returned to work within 13.4 weeks (n = 48). Patients who had a re-rupture returned to work in 8.75 weeks (n = 4), compared to 5.5 weeks for those without a re-rupture (n = 72). However, with only two patients in the re-rupture group returning to work, a larger sample size is required to draw further conclusions about return-to-work timelines after re-rupture (Figure 1).

The dataset revealed that, on average, patients under the physiotherapy-led pathway returned to sports activities in about 29.9 weeks. The extended period to return to sports compared to work highlights the necessity for a fully rehabilitated tendon to withstand the stresses of sports activities. The median time to return to sporting activity was 28 weeks, with the proximity to the mean suggesting symmetrical data distribution around the central value and consistency across the sample.

Thromboprophylaxis was prescribed for 93.5% (n = 71) of patients with ATR. The data revealed a low incidence rate of DVT with only 4% (n = 3) of patients experiencing this complication post rupture. This low percentage is significant, demonstrating that while DVT remains a concern, the risk with ATR under this pathway is minimal. The successful minimization of DVT can be attributed to factors such as early mobilization strategies or the selective use of pharmacological prophylaxis where appropriate. Most patients in this cohort (93.4%) did not receive any medication, showcasing a diverse approach to DVT prophylaxis. This indicates individualized prophylaxis based on risk factors such as age and travel, rather than broadly applying DVT prevention.

## Discussion

We are now seeing increasing numbers of patients presenting to the hospital with ATR due to the ageing population and the increased uptake of sporting activity by middle-aged individuals [15]. Due to the ever-increasing toll on NHS services post pandemic and the rise in waiting lists, it is important to find new effective strategies to manage workload and improve patient experience and safety. This includes a re-organization of service delivery and the introduction of more physiotherapy or allied health professional-led treatment pathways [17].

This retrospective study presents a comprehensive overview of outcomes and demographics of patients treated along the physiotherapy-led treatment pathway at our district general hospital for ATR. It shows that the majority of patients presenting were in the 40-60 years age group, which is consistent with the literature [9].

The outcome data, showing a low 5% re-rupture rate, suggests the physiotherapy-led rehab protocol is effective in preventing further complications or injury recurrence. This is consistent with the 5.7% noted in those treated non-operatively in the literature [10]. The gender split in re-ruptures, with three male and one female affected, suggests a potential gender disparity, but further research and a larger sample size are required for conclusive results. Furthermore, only 14.5% of patients needed escalation to a consultant for assessment, suggesting that the initial physiotherapy-led management plan was largely effective. This highlights physiotherapists’ proficiency in treating ATR and suggests the potential for cost reduction and their benefit in alleviating pressure on specialist services by effectively managing these cases at the primary care level [18].

In addition, while over half of the patients resumed driving within 10 weeks post injury, those with complete ruptures experienced a longer delay, indicating the severity and extended rehabilitation for complete ruptures. This emphasizes the necessity of personalized rehabilitation protocols considering injury severity, possibly integrating functional activities to aid in resuming driving.

The 4% incidence of DVT in ATR patients receiving physiotherapy reflects effective risk mitigation within current management protocols. Factors like early mobilization likely contribute to this success by enhancing venous return. The selective use of pharmacological DVT prophylaxis reflects a patient-centred strategy, emphasizing individual risk factors. This approach advocates for continued risk assessment-based prophylaxis for DVT, while also stressing the need for symptom monitoring, especially in patients with additional risk factors [19].

This study, though informative, has limitations affecting the ability to draw definitive conclusions and generalize findings. The dataset's relatively small size (n = 76) may limit its representation for the broader population with ATR. The retrospective, observational study design limits the ability to draw causal inferences from the data. Prospective studies or randomized controlled trials are required for stronger evidence for physiotherapy-led management efficacy. Without a control group receiving different treatments, attributing positive outcomes solely to the physiotherapy-led approach is challenging due to potential confounding factors. Lastly, the study did not consider geographic and cultural contexts, which can impact patient access to care and treatment protocol adherence. These factors caution against drawing definitive conclusions from the study and underscore the necessity for further research with stricter methodologies.

## Conclusions

This retrospective study offers insight into physiotherapy-led ATR management at a district general hospital, evaluating treatment results and challenges. The findings suggest that a conservative approach is effective in promoting patient recovery, with low re-rupture rates and successful return-to-function outcomes. While limitations such as sample size and retrospective design exist, the findings underscore the need for tailored rehabilitation protocols and continued research to optimize patient care in ATR management.
